# An Explanation for the Role of the Amygdala in Aesthetic Judgments

**DOI:** 10.3389/fnhum.2017.00080

**Published:** 2017-03-02

**Authors:** Richard H. A. H. Jacobs, Frans W. Cornelissen

**Affiliations:** ^1^Donders Institute for Brain, Cognition and Behaviour, Radboud University NijmegenNijmegen, Netherlands; ^2^Laboratory for Experimental Ophthalmology, University Medical Center Groningen, University of GroningenGroningen, Netherlands

**Keywords:** neuroaesthetics, aesthetic judgment, amygdala, feature-based attention, beauty, functional magnetic resonance imaging

## Abstract

It has been proposed that the top-down guidance of feature-based attention is the basis for the involvement of the amygdala in various tasks requiring emotional decision-making (Jacobs et al., 2012a). Aesthetic judgments are correlated with particular visual features and can be considered emotional in nature (Jacobs et al., 2016). Moreover, we have previously shown that various aesthetic judgments result in observers preferentially attending to different visual features (Jacobs et al., 2010). Here, we argue that—together—this explains why the amygdalae become active during aesthetic judgments of visual materials. We discuss potential implications and predictions of this theory that can be tested experimentally.

## Introduction

One of the goals of aesthetic neuroscience is to unravel the brain mechanisms involved in the judgment of beauty. In a recent study (Jacobs et al., 2012b)—discussed in more detail below—we found—among other results—that the amygdala became more active during beauty judgments compared to a number of other judgments, implying a specific involvement of the amygdalae in making beauty judgments. Based on the amygdalar involvement in emotion and reward processing (Murray, 2007) and its activation differentiating between faces of different valence (Killgore and Yurgelun-Todd, 2004), we anticipated an amygdalar role in aesthetics—e.g., in the sense of its response being different for ugly and beautiful stimuli. Nevertheless, the amygdalar differentiation between aesthetic and non-aesthetic judgments came somewhat as a surprise. At the time, we stated the notion that “*the amygdalar role in assessing beauty may consist of guiding attention to the features that are relevant for making the beauty or other evaluative assessments*”. This notion was based on our proposal that the amygdala is involved in the top-down guidance of feature-based attention (Jacobs et al., 2012a). Here, our intention is to develop this notion into a testable theory that explains why and how the amygdala plays a critical role in aesthetic judgment.

## Amygdalae Guide Top-Down Feature-Based Attention

The amygdalae are among the most heavily studied brain centers in neuroscience, and are commonly viewed as emotion processors. Despite decades of research, their precise function remains elusive and is the subject of ongoing debate (Whalen and Phelps, 2009). After initial reports that the amygdalae function as fear processors, more recent studies indicate a broader function, encompassing also the processing of other emotions. One of the most revealing findings in recent years indicates that amygdalar damage leads to disrupted scanning of expressive faces, but not to impaired emotion recognition *per se* (Adolphs et al., 2005). Other studies have implicated the amygdala in orienting, eye movements and attention (Bancaud et al., 1966; Anderson and Phelps, 2001; Ohrmann et al., 2007; van Reekum et al., 2007; Cunningham et al., 2008; Carlson et al., 2009), usually directed at emotionally relevant information. Recent findings suggest that attention-attracting effects of expressive faces are based on constitutive elements. Facial elements, such as eye-whites and the white of exposed teeth in photographs of faces, or orthogonally oriented lines in schematic faces, or downward-pointing V-shapes, resembling a frown, have been shown to attract attention (Horstmann et al., 2006; Larson et al., 2007; Calvo and Nummenmaa, 2008; Calvo and Marrero, 2009; Coelho et al., 2010). The amygdala may be particularly sensitive to low-spatial frequency information (Vuilleumier et al., 2003; Winston et al., 2003; for a negative finding, see Morawetz et al., 2011). This appears in several measures, such as costs and benefits in search times, or in costs in time to identify a centrally presented facial expression flanked by other facial expressions. These results suggest that attention to emotional information is based on features defining the relevant elements. Combined with the finding that amygdalar activation to expressive faces is associated with the efficiency of finding expressive faces (Ohrmann et al., 2007), these findings suggest that the amygdala may direct attention to these features. Moreover, the amygdala responds to features such as angularity (Bar and Neta, 2007). Hence, we have previously proposed that the amygdalar function is to guide feature-based attention (Jacobs et al., 2012a). Feature-based attention consists of attending to some features over others, even when they overlap spatially. We consider features to be dimensions along which visual or other information can vary. For example, colors such as yellow are features because pictures differ in the amount of yellowness contained in them. The same goes for spatial frequencies and for more complex features such as entropy. We explicitly reject the idea that elements, such as noses, eyes and ears in faces, are features, although such elements can only stand out because of a discontinuation of the feature values along their borders. A very simple example of feature-based attention would be attending to the color rather than the orientation of a bar, but there could be many types of features to which attention might be directed. This attention could be used to search for items when they are not in the focus of attention, or to enhance processing of certain stimulus aspects over others, even when residing in the focus of attention. Taken together, these findings suggest to us that the amygdala plays a key role in selecting the visual features that are relevant for making decisions. That this concerns emotional decisions in particular is suggested by the presence of higher amygdala activation to explicitly emotional tasks than to other tasks, as was shown in a meta-analysis (Fusar-Poli et al., 2009).

## Aesthetic Judgments Are Correlated with the Presence of Particular Visual Features

It is a long held belief that there may exist universally appreciated stimulus properties and regularities in what people consider beautiful (Konecni, 2012). Despite this, we are aware of only a small number of studies into the actual relationships between image features and beauty ratings. Aesthetics research has often focused on stimuli such as paintings or faces, in which featural information is hard to control, and such control is usually not even attempted. Several studies have found relationships between preference and color features (Ball, 1965; Valdez and Mehrabian, 1994). Still, the number of studies investigating preferences for features or texture (Soen et al., 1987; Kawamoto and Soen, 1993; Aks and Sprott, 1996; Schira, 2003) is greatly exceeded by the vast amount of research that has been devoted to understanding the affective responses to objects. Nevertheless, there are indications in the literature that texture—a pattern without a single object outline and which can be thought of as consisting of repetitive arrangement of an element or a number of elements, which can be captured in summary statistics, i.e., in featural information—may have an impact on preference. Such indications come from studies investigating the relationship between preference and the fractal dimension (Aks and Sprott, 1996; Bies et al., 2016), entropy (Stamps, 2002), spatial frequency content (Soen et al., 1987; Kawamoto and Soen, 1993; Schira, 2003) or certain colors (Valdez and Mehrabian, 1994; Jacobs et al., 2010; Schloss and Palmer, 2011) of stimuli, as well as from work showing that paintings contain certain spatial frequency characteristics (Redies et al., 2007; Graham and Redies, 2010). Moreover, texture influences facial attractiveness to a large extent (Jones et al., 2004). In line with the reported relationship between spatial frequencies and beauty ratings, the brain responses to affective stimuli—such as expressive faces—depend on the frequency bands present in the stimulus (Vuilleumier et al., 2003; Holmes et al., 2005; Alorda et al., 2007; Delplanque et al., 2007). Finally, Jacobs et al. (2016), have shown that there is a moderately strong correlation between the beauty ratings assigned by observers to certain textures, and a number of computationally derived visual features (e.g., intensity variation, spatial frequencies, luminance and color) contained in the texture images.

To summarize, there are strong indications that the degree to which certain relevant features are present in an image affects the likelihood that observers will rate it as being beautiful.

## Aesthetic Judgment Results in Observers Preferentially Attending to Specific Visual Features

In his seminal work, Buswell (1935) demonstrated that fixation locations on scenes differ according to the questions the observer had to answer. This finding has been confirmed many times (Yarbus, 1967; Lipps and Pelz, 2004; Rothkopf et al., 2007; DeAngelus and Pelz, 2009; Underwood et al., 2009). As Buswell’s questions related to information that was present in different parts of the pictures, his finding may not appear too surprising, yet it was the first formal demonstration of task effects on the guidance of the eyes. Subsequently, eye movements have been used as a tool by researchers to reveal covert perceptual and cognitive processes that underlie the perception and aesthetic evaluation of artworks (for a review see Nodine et al., 2003). Most actual artworks are inhomogeneous in terms of both space and features, which renders them interesting, but also less suitable for examining the role of feature-based attention. For this reason, textures—that by definition are spatially more homogeneous in their element distributions—are a more suitable type of stimulus to study this. In an eye movement experiment (Jacobs et al., 2010), we asked participants to judge textures on their beauty and roughness, while their gaze-behavior was recorded. The similarity in the overall spatial distribution of attention suggested that differences in the guidance of attention are non-spatial, and presumably feature-driven. Nevertheless, during the beauty judgment, participants tended to fixate on patches that were richer in color information. A study employing landscapes and portraits as stimuli (Massaro et al., 2012) confirmed the importance of color information for aesthetic judgments. In this study, observers made more fixations to colorful images than to black-and-white images, but only during aesthetic judgments and not when they judged for the presence of movement in the same stimuli. These findings further supported the idea that the differences in the distribution of attention—as evident from the distributions of fixations—were feature-driven. These results showed differences in eye movements accompanying aesthetic and non-aesthetic judgments about visual textures as well as evidence for attending to different features during either task. In summary, eye-movement studies suggest that observers select different features for scrutiny during aesthetic compared to during non-aesthetic judgments.

## The Amygdala Are Activated during Feature- but Not Space-Based Attention

Mohanty et al. (2009) found that the amygdala activates when cues about the emotional (i.e., angry or neutral) nature of a target—a tilted face among upright faces—were presented, but not when similar cues indicated the location of the target among the upright faces. We interpret this result as support for the notion that the amygdala is involved in feature-based and not space-based attention. In our interpretation, presenting the emotional cue primed neurons sensitive to the features that enable discrimination between neutral and angry faces, which in our view is the neural basis of feature-based attention. Once selected, spatial attention was engaged to limit target search to the cued faces, as in the spatial cueing task.

## The Amygdala Are Activated during Aesthetic Judgments

**Figure 1:**
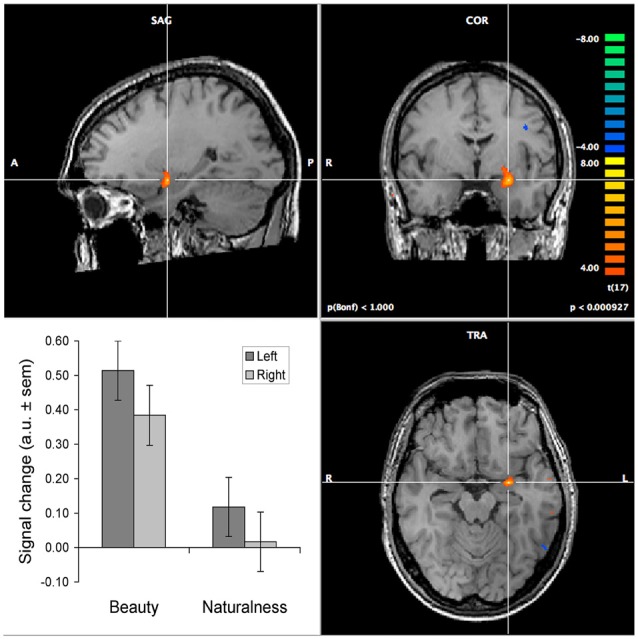
**Selectively increased activation of the amygdalae during the judgment of texture beauty and naturalness.** The images show the increase in activation relative to the judgment of texture roughness. The activation of the left amygdala is centered around Talairach coordinates −25, −3, −15. The bar graph shows the results of a region-of-interest analysis, performed separately on the left and right amygdalae, contrasting both beauty and naturalness judgments to roughness judgments (the baseline). This yielded highly significant effects for beauty compared to both roughness and naturalness, in both hemispheres (all *p* < 0.0001), but not between naturalness and roughness. The brain images show activation in the left amygdala for the beauty minus roughness contrast, at a *p*-value of 0.001 (for illustrative purposes uncorrected for multiple comparisons). The colormap indicates *t*-values exceeding this threshold, with orange-to-yellow indicating higher activation during the beauty judgment and blue-to-green indicating higher activation during the roughness judgment. Details on the experimental procedure and results for other brain regions can be found in Jacobs et al. (2012b).

Neuroaesthetics has primarily tended to focus on the neural correlates of observing beautiful stimuli compared to neutral or ugly stimuli. This has the potential of confusing stimulus property-driven and task (i.e., internal state) related activations. Evidence for amygdalar involvement in aesthetic vs. non-aesthetic judgments for the same set of stimuli does not suffer from such confounds. Exactly such a finding was reported by Di Dio et al. (2007), for judgments of aesthetics in comparison to proportion judgments and passive observation. However, this experiment potentially suffered from a confound between the proportion and aesthetics judgment. We addressed this issue by using orthogonal judgments, as determined by factor analysis (Jacobs et al., 2016). In an fMRI experiment in which participants judged the same set of visual textures on their beauty, naturalness and roughness, we found that the amygdala—among other regions—was more strongly activated during beauty than during non-aesthetic judgments (see Figure 1), which is most notable for the stimuli judged as beautiful (see Jacobs et al., 2012b). This result shows that there is a relationship between aesthetic judgment and activity level of the amygdala. First of all, these results imply that the amygdala is part of a network that is involved in deliberate aesthetic assessment. Moreover, participants judged texture stimuli, which tend to look similar everywhere, such that effects of shifting spatial attention are minimal. On the premise that selective attention comes in two flavors—feature- and space-based attention—this implies that the amygdalar involvement in the guidance of attention when judging texture-stimuli is likely to be feature- rather than space-based.

We argue this is so, because the amygdala plays an essential role during emotional decision-making, which an aesthetic judgment is. Its task is the selection of non-spatial information (visual features) that is critically required in making the aesthetic (emotional) decision. Importantly, this is even so, when the stimulus is not emotional by nature (e.g., a texture rather than a face).

In summary, findings indicate that the amygdala is involved in aesthetic judgments, and we suspect in emotional decision-making in general. Since we employed visual textures as stimuli, the role of spatial attention is minimal, which in our opinion only leaves room for feature-based guidance of attention.

## Summary of the Hypothesis

Our hypothesis is that the amygdala plays a critical role in aesthetic judgments because it guides attention to the relevant visual features whose assessment is required to make the—emotionally tinged—judgment.

In particular, this function consists of weighting the prior expectation (baseline activation levels) of neurons coding for specific feature dimensions (e.g., orientation or color) that the observer has learned to associate with specific judgments.

## Questions and Predictions

Our hypothesis raises a number of questions and licenses various predictions and recommendations for experiments on how to test these.

## Amygdalar Involvement in Various Aesthetic Judgments

In most of our experiments, we have equated aesthetic judgment with the judgment of beauty. This choice was motivated by the need to keep experiments simple, as well as our finding that the judgment of beauty aligned well with the “evaluative” axis of a two-dimensional space—estimated using principal component analysis—that could explain a substantial amount of variance in a range of judgments (the other “descriptive” axis aligned well with roughness; Jacobs et al., 2016). Therefore, a first prediction is that the amygdalae primarily become active during judgments that align well with the evaluative axis in this space, and not (or much less) during judgments that align with the descriptive axis. The evaluative axis involves judgments such as those for colorfulness, elegance, interestingness. When comparing two judgments that both align with this axis (e.g., symmetry and beauty judgments) we predict no amygdala activation (congruent with the results of Jacobsen et al., 2006). Moreover, we predict that judgments that are primarily feature-based (e.g., judgments based on color information) will activate the amygdala but not those that are primarily space-based (e.g., judging the composition of paintings or the use of the golden ratio). This could explain the absence of amygdala activation in a study that compared an aesthetic condition to a “detached” condition (Cupchik et al., 2009). In this study, participants were instructed to pay attention to spatial composition, color as well as other features. Besides that it was unclear to what extent observers adhered to this instruction (no explicit responses were collected), they may have based their (internal) evaluations primarily on the spatial composition of the artworks.

Our theory predicts differential activations for different tasks, not for different ratings. Hence, it makes no specific predictions for studies contrasting beautiful to ugly (or positive to negative) ratings. Likewise, deciding on beauty or ugliness most likely involves judging on the basis of the same feature dimensions and is thus predicted to similarly engage the amygdala.

## Could One Still Make Aesthetic Judgments without An Amygdala?

Strong supporting evidence for our theory could come from studying patients with damaged amygdalae (e.g., due to Urbach-Wiethe disease). According to our theory, a damaged amygdala should severely disrupt the guidance of attention to the relevant features for making an aesthetic judgment. It will be next to impossible to compare judgments for the same stimuli before and after the occurrence of amygdalar damage. Although animals may show signs of aesthetic appreciation, we consider it important to study this faculty in humans. How would aesthetic appreciation appear in behavioral measures? Presumably, participants would still be able to press buttons to indicate some kind of evaluation. Given that with evaluations of beauty or liking there is no ground truth to which the evaluations can be compared, these cannot easily be classified as right or wrong. Nevertheless, we predict that patients with amygdalar damage may show strongly deviant judgments compared to healthy participants and would likely be less consistent in their evaluations of repeated stimuli. However, before this can be reliably assessed, some experimental issues need to be resolved. Patients would probably still be able to recognize the stimuli which they had seen before, and also remember their previous answers. Participants are likely to strive for consistency in their evaluations (Höfel and Jacobsen, 2003), so in order to demonstrate an increased inconsistency it would be necessary to let them forget their previous scores. This could be achieved by having them evaluate so many stimuli that they cannot keep track of their evaluations, or by distracting them in dual-task settings or with filler-tasks. Even under such circumstances an increased variability is not assured, since it is possible that the patients base their judgment on the information available during the initial fixation presumably in the middle of the stimulus as would be predicted from the scanning patterns of Adolphs’ patient on faces (Adolphs et al., 2005). Alternatively, the judgment may be based on systematic spatial scanning of the entire stimulus. The latter strategy could be prevented by brief presentation durations. To avoid consistency of the evaluations by their being based on the information at central fixation, we would propose randomly offsetting the stimuli away from the screen’s center or presenting the same stimuli in various cut-outs.

Perhaps an easier way to test the effects of amygdalar damage on aesthetic evaluations is to examine patients’ eye movements as a proxy to studying task-dependent attentional guidance. Based on our finding that observers fixate more colorful stimulus patches during aesthetic than during non-aesthetic judgments, we predict that the presence of this difference depends on intact amygdalae. We foresee three possible outcomes: (i) the damaged amygdala results in a steady fixation on the stimulus center (as occurs during the judgment of emotional faces; Adolphs et al., 2005); (ii) if saccades do occur, the bias to fixate on specific features (e.g., colorful patches) during aesthetic evaluations will disappear. Also, we expect that the previously observed differences in the number of fixations and the length of saccades for healthy observers (Jacobs et al., 2010) will be absent in patients; and (iii) observers may employ space-based rather than feature-guided fixation patterns. In the absence of feature-based guidance of attention, we believe patients could resort to strategies such as serially scanning visual scenes (e.g., in a reading-like fashion). We believe that such strategies are based on spatial information, and could therefore survive amygdalar damage. The patient studied by Adolphs et al. (2005) was able to categorize emotional expressions when told to focus on the eyes—which in the fixed configuration of a face could then be solved in a spatial manner. Regardless of which of the above options will turn out to be true, task-dependent differences in fixation count or duration and saccade length should be absent in patients.

## Individual Differences in Aesthetic Appreciation

Different observers may use different features and also weigh them differently when judging aesthetically, thus facilitating a large degree of individual and cultural variation in the aesthetic appreciation of various art forms and stimuli. Similarly, expert observers may use different features compared to novices when judging aesthetics. However, irrespective of what features observers ultimately use, we predict very similar task-dependent differences in amygdalar activation within observers, as we expect that the amygdala will have learned to guide attention to whatever feature is relevant for the task at hand.

## A Role for the Amygdala Also in Non-Visual Aesthetic Judgments?

The amygdalae are involved in orienting to smells and sounds (Ursin and Kaada, 1960a,b; Ganzha, 1986; Zald and Pardo, 1997; Sobel et al., 1999) as much as in orienting to visual stimuli. Hence, we predict that aesthetic evaluation of music, food, mathematical formulae (Zeki et al., 2014) and maybe even of sculptures or surfaces that can only be felt (e.g., in complete darkness) will also (besides visual textures and shapes) differentially involve the amygdala. However, tracking eye movements may not always make much sense for such stimuli. Hence, new behavioral or imaging indices would have to be found. Exploration patterns in the haptic evaluation of surfaces may provide clues, in which case we predict different exploration patterns for aesthetic compared to other types of judgment, similar to what we found for the visual exploration of textures. In case of music, attentional guidance to different frequency patterns may become evident from distinct activations in tonotopic maps in the auditory cortex (Langers and van Dijk, 2012). An important consideration when designing experimental procedures for neuro-imaging experiments is that the activations for amygdalar involvement during aesthetic judgments should be compared to the activations found to the same stimuli but in a different task condition. This enables excluding stimulus- or valence-driven activations (Jacobs et al., 2012b).

## Conclusion

Starting from our recent proposal that the top-down guidance of feature-based attention is the basis for the involvement of the amygdala in various tasks requiring emotional decision-making (Jacobs et al., 2012a) we have argued that this notion may also explain the involvement of the amygdala in aesthetic judgments. Based on our theory, we have developed a number of testable predictions ranging from the consequences of amygdala damage on aesthetic judgments to the prediction that there is a role for the amygdala also in various non-visual aesthetic judgments.

Testing these predictions is important to our understanding of the mechanisms underlying aesthetic judgments. Particularly, it will address issues such as the relationship between aesthetic and other emotionally tinged judgments, the interplay of bottom-up and top-down influences in processing of aesthetic materials, and the nature of different forms of attention.

## Author Contributions

RHAHJ and FWC contributed to the writing of the manuscript, and to the acquisition and analysis of reported data.

## Funding

This work was funded by the University Medical Center Groningen.

## Conflict of Interest Statement

The authors declare that the research was conducted in the absence of any commercial or financial relationships that could be construed as a potential conflict of interest.
